# Assessment of Serum Vitamin D Levels in Patients with Vitiligo in Jordan: A Case-Control Study

**DOI:** 10.1155/2019/2048409

**Published:** 2019-10-10

**Authors:** Diala M. Alshiyab, Firas A. Al-qarqaz, Leen H. Heis, Jihan M. Muhaidat, Wlla S. Eddin, Ausama A. Atwan

**Affiliations:** ^1^Department of Dermatology, Faculty of Medicine, Jordan University of Science and Technology, Jordan; ^2^Department of Dermatology, Royal Gwent Hospital, Newport, UK

## Abstract

**Background:**

Low vitamin D serum levels have been associated with many autoimmune disorders and several other skin diseases. Vitiligo is an autoimmune disease characterized by destruction of melanocytes by immune mechanisms. Melanocytes express vitamin D receptors, and their function can be affected by vitamin D status.

**Objectives:**

The main objective of this study is to compare vitamin D levels in patients with vitiligo vs normal population and whether vitamin D deficiency is associated with vitiligo.

**Methods:**

A case-control study was conducted. 100 vitiligo patients and 100 as controls were included in this study. Serum vitamin D level was measured for both vitiligo patients and controls, results were compared, and statistical analysis was done to compare the results.

**Results:**

The median age of vitiligo cases was 23 years (ranges, 2–80). 58% of vitiligo patients were females. The median vitamin D level was not significantly different between the two groups (vitiligo = 14.1 (IQR 9.9–20.4) vs control = 16.5 (IQR 10.3–25.3) (*P*=0.28)). Most vitiligo cases and controls were found to have low levels of vitamin D (either insufficient 20–30 ng/mL or low <20 ng/mL).

**Conclusions:**

There was no significant difference in vitamin D levels in vitiligo patients compared to controls. However, vitamin D levels were generally low in both groups.

## 1. Introduction

Vitiligo affects about 2% of the world population irrespective of the skin type, age, and sex [1, 2]. It is characterized by skin depigmentation as a result of destruction of melanocytes in the affected areas. Although the pathogenesis is not fully understood, it is considered as an autoimmune disease. Association with other autoimmune conditions such as pernicious anaemia, alopecia areata, systemic lupus, and thyroid disease has been established [2–4]. Genetic factors play an important factor in pathogenesis. Several studies including studies on Jordanian population have implicated several genes in the pathogenesis of vitiligo [5, 6].

Vitiligo has a great psychological and social impact on affected people especially females or if on exposed sites and more so in people of color (POC) [7].

Vitamin D3 is an essential vitamin for humans. The majority of its active form is obtained through activation of the pre-vitamin D3 formed in the skin after sun exposure particularly UVB (290–320 nm). Diet is only a minor source for this vitamin. This vitamin has a significant role in immunity (innate and adaptive), calcium regulation, and melanin synthesis; in addition, many diseases have been associated with reduced vitamin D levels [3]. Melanocytes express receptors for vitamin D which may indicate a possible role for vitamin D in regulation of melanocyte function [8]. Furthermore, topical vitamin D (calcipotriol) has been used as treatment for patients with vitiligo as it might help in preventing destruction of melanocytes [8].

The aim of this study is to see whether vitamin D levels in patients with vitiligo are lower than normal controls in this population of colored skin.

## 2. Materials and Methods

A case-control study was conducted. 100 vitiligo patients attending dermatology clinics at King Abdullah University Hospital, Irbid, North of Jordan, were included between May and December 2018. Full history and examination were done, and the diagnosis was confirmed clinically and by using Wood's light examination. In addition, 100 controls, age and sex matched, were included for comparison.

Patients with vitiligo (generalized, focal, or segmental), normal thyroid function tests, and serum vitamin B12 were included in the study. In addition, clinical details of vitiligo were obtained including patient demographics, duration of vitiligo, and current and previous treatment. Patients were excluded if there was a history of autoimmune disease, abnormal thyroid function, or serum B12 level. Also, patients with vitiligo were excluded if they had phototherapy treatment or vitamin D supplements within the last year. Controls included healthy visitors or patients coming to dermatology clinics for other reasons. Controls were excluded if they had a history of any autoimmune disease, abnormal thyroid function tests, or serum B12 levels or intake of vitamin D supplements. The serum levels of 25-hydroxyvitamin D3 were measured in the laboratory of King Abdullah University Hospital using the Elecsys Vitamin D total II cobas by Roche. In accordance with the vitamin D level, the status of vitamin D was classified into the following categories: normal (>30 ng/mL), insufficient (20–30 ng/mL), and low (<20 ng/mL) using the reference values at the lab where samples were analysed. All patients signed a consent form before participating in this study.

The study was approved by the local IRB committee (32-106/2017).

### 2.1. Statistical Analysis

Statistical analysis was performed using the SPSS software version 22. The median and interquartile range (IQR) were provided for quantitative variables. A *P* value <0.05 was considered statistically significant.

## 3. Results

A total of 100 vitiligo patients and 100 controls were included in the study. The median age for vitiligo patients was 23 years (2–80 yrs) with a female : male ratio of 1.38 : 1. For controls, the median age was 31.5 years (2–74 yrs) with a female : male ratio of 1.85 : 1, and there was no statistical significant difference in the main demographic features for both groups. The main demographic features of the study population are shown in Table 1.

In vitiligo patients, the median level of vitamin D was 14.1 ng/mL, while in controls it was 16.5 ng/mL (IQR 10.3–25.3) (*P*=0.28), and there was no statistical significant difference between vitamin D levels in patients with vitiligo compared to controls (Table 1).

The vitamin D levels in vitiligo patients were low in 74% and insufficient in 13%, while in controls 59% had low levels and 25% had insufficient levels.

We tried to look for association between vitamin D levels and clinical characteristics of vitiligo including disease duration, type of vitiligo (generalized vs localized and segmental subtypes), and relation to vitiligo treatment; however, we could not find any statistically significant relation between these features and vitamin D levels, and this indicates that none of these variables in vitiligo patients could be linked to the levels of vitamin D (Table 2).

## 4. Discussion

Vitamin D is an essential hormone synthesized in the skin, and its deficiency has been associated with several conditions including immune, metabolic, and pigmentary disorders. It was shown that vitamin D increases melanogenesis and the tyrosinase content of cultured human melanocytes [8]. Additionally, it increases the number of L-3,4-dihydroxyphenylalanine- (DOPA-) positive melanocytes after 1,25 (OH)_2_D_3_ treatment in the primary neural crest cell (NCC) culture. In a study by Watabe et al., 1,25(OH)_2_D_3_ was found to induce endothelin B receptor (EDNRB) expression in immature melanocytes such as NCC-melb4 cells, but not in mature melanocytes such as NCC-melan5 cells. A melanocyte stem cell-like subpopulation is thought to be present in the bulge region of hair follicles, and it is thought that vitamin D may act to induce immature melanocytes in hair follicles to produce melanin by stimulating their differentiation and their expression of EDNRB [9]. The relationship of the vitamin D sreceptor (VDR) in vitiligo has been studied, and the level of VDR ApaI locus was found to be increased in vitiligo patients [10]. According to a study by Sauer et al., 1alpha, 25-dihydroxyvitamin D3 protects human melanocytes from apoptosis by the formation of sphingosine-1-phosphate [11]. The role of vitamin D in protecting human melanocytes against oxidative damage has been identified in other studies [12].

Several studies were conducted to investigate the relation between vitiligo and vitamin D [10, 13–17]; however, the results of these studies were nonconfirmatory. In a study by Beheshti et al., serum vitamin D levels were shown to be low in vitiligo patients; however, no control group was included [13]. A meta-analysis that included 17 studies showed that vitamin D deficiency was positively associated with the incidence of vitiligo [10]. A Chinese study included 114 children with vitiligo who show lower vitamin D level and was associated with the onset of vitiligo [14]. On the contrary, other studies found no significant differences in vitamin D levels among the vitiligo patient and the control [15–17]; however, these studies were conducted on a small number of patients.

In the current study, the levels of vitamin D were lower than normal; however, this was comparable to the control group. In our population, the prevalence of vitamin D deficiency is very high [18–20], and the possible explanations for this deficiency is related to the more conservative clothing style (due to cultural and religious reasons) especially in Jordanian females wearing hijab and covering their bodies almost completely which could interfere with vitamin D synthesis. The dark skin color in this population could be another contributing factor since the majority of our population has darker skin colors, and pigmented skin is a known risk factor for vitamin D deficiency as melanin filters UV radiation. A study has shown that white girls have higher vitamin D levels than black girls in the United States [21]. Another study from the Southeastern United States showed low vitamin D status is prevalent among adolescents living in a year-round sunny climate, particularly among black youths [22]. This high prevalence of vitamin D deficiency may make detection of difference in vitiligo patients more difficult, and a very large number of patients and controls may be needed to show such differences should they exist. The complex and multifactorial pathogenesis of vitiligois is another important consideration. For example, in our population with higher consanguinity rates, genetic factors may have more important contribution to pathogenesis.

## 5. Conclusion

Vitamin D levels were low in both vitiligo and controls of this population. There was no significant difference in the levels of vitamin D in both groups.

## Figures and Tables

**Table 1:** Main demographics of the study and vitamin D levels.

	Vitiligo, *N* (%) mean	Controls	*P* value
Age	Average 23 years (2–82 years)	Average 31.5 years (2–74 years)	*P*=0.057
Sex	58 females, 42 males	65 females, 35 males	
Normal vitamin D	(13)% Mean 34.9	16% Mean 41.89714286	*P*=0.078
Vitamin D insufficiency	13% Mean 25.1	25% Mean 24.9	*P*=0.82
Vitamin D deficiency	74% Mean 11.9	59% Mean 11.2	*P*=0.37
Median vitamin D	14.1	16.5	*P*=0.28

**Table 2:** Relation of vitiligo characteristics and duration to vitamin D levels.

Vitamin D level	Average duration (years)	Type of vitiligo (localized/segmental vs. generalized)	Treatment
Deficient (*N* = 74)	2.95	*G*=51	N/T = 49
		L/S = 23	P/T = 25
Insufficient (*N* = 13)	3	*G*=9	N/T = 10
		L/S = 4	PH/T = 3
Normal (*N* = 13)	2.89	*G*=10	N/T = 11
		L/S = 3	
*P* value	^*∗*^ *P*=0.21	^*∗*^ *P*=0.42	^*∗*^ *P*=0.39

*G*, generalized type of vitiligo; L/S, localized/segmental types; N, no treatment used; T, topical treatment; PH, phototherapy. ^*∗*^*P* value shown for the vitamin D level and duration, type of vitiligo, and treatment modality.

## Data Availability

Data used in the article are available on request from the corresponding author.

## Additional Points

*Limitations.* The results of this study could be limited by the relatively small number of patients and controls. High prevalence of vitamin D deficiency in this population is another possible limitation to this study.
